# Reassessing Estrogen Receptor Expression Thresholds for Breast Cancer Prognosis in HER2-negative Patients Using Shape Restricted Modeling

**DOI:** 10.21203/rs.3.rs-3466989/v1

**Published:** 2023-10-28

**Authors:** Wenli Dong, Takeo Fujii, Jing Ning, Toshiaki Iwase, Jing Qin, Naoto T Ueno, Yu Shen

**Affiliations:** University of Texas MD Anderson Cancer Center Division of Quantitative Sciences; National Cancer Institute Center for Cancer Research; University of Texas MD Anderson Cancer Center Division of Quantitative Sciences; University of Hawai’i Cancer Center; National Institute of Allergy and Infectious Diseases; Cancer Research Center of Hawaii: University of Hawai’i Cancer Center; University of Texas MD Anderson Cancer Center Division of Quantitative Sciences

**Keywords:** Estrogen receptor, Threshold, Survival, Modelling, Endocrine therapy

## Abstract

**Purpose:**

To assess the dynamic link between continuous estrogen receptor (ER) expression and long-term clinical outcomes in non-metastatic breast cancer and to identify the ideal cutoff value for ER expression to optimize endocrine therapy use.

**Methods:**

The study included 3055 female patients with stage II or III HER2-negative breast cancer. The primary outcomes were time to recurrence or death (TTR) and overall survival (OS). We used a novel shape-restricted Cox model to determine the desirable ER expression cutoff to predict breast cancer prognoses. Our novel model allows ER as a continuous variable, utilizing a flexible monotone-shaped Cox regression to assess its association with survival outcomes holistically.

**Results:**

The shape-restricted Cox model identified 10% ER as the preferred cutoff to predict TTR. The finding was confirmed by the log-rank test and standard Cox model that patients with ER ≥ 10% had TTR benefit over ER < 10% (log-rank p < 0.001). No OS or TTR benefit of adjuvant endocrine therapy was observed in patients with 1% ≤ ER < 10% (HR 0.877, 95% CI 0.481–1.600, p = 0.668 for TTR and HR 0.698, 95% CI 0.337–1.446, p = 0.333 for OS).

**Conclusions:**

Using the shape-restricted Cox model, this study suggests a potential preferred threshold of 10% for predicting TTR. The findings could assist physicians in effectively weighing the benefits and risks of adjuvant endocrine therapy for patients with ER < 10% disease, particularly in cases involving severe adverse events. Further prospective studies are warranted to validate the recommended cutoff value.

## Introduction

The ideal cutoff values for estrogen and progesterone receptors (ER and PR, respectively) to distinguish the efficacy of endocrine treatment in breast cancer have been long debated [1–5]. ER and PR positivity was determined at 10% until 2010, when the American Society of Clinical Oncology/College of American Pathologists (ASCO/CAP) published their updated guidelines [6]. Therein, ER and PR expression < 1% were considered ER/PR-negative. However, we previously reported that 10% of ER is an ideal threshold to predict the pathological complete response (pCR) to the neo-adjuvant chemotherapy (NACT), time-to-recurrence or death, showing that patients with ER < 1% and patients with ER 1%–10% had similar short-term clinical outcomes (pCR rates of 26.3% and 28.1%, respectively) among patients with HER2-negative breast cancer; only patients with ER > 10% had survival benefits from adjuvant hormonal therapies [7]. Another study, which involved 9639 patients with primary breast carcinoma treated at The University of Texas MD Anderson Cancer Center from 1990 to 2011, demonstrated that, regardless of HER2 status, primary breast carcinoma patients with an ER expression of 1%–9% had similar recurrence-free survival (RFS) to those with an ER expression of < 1% (hazard ratio (HR) = 1.2, 95% confidence interval (CI) of HR = 0.9–1.7, p = 0.2), and both groups had significantly worse RFS than those with an ER expression of ≥ 10% [8]. This suggests that an ER expression of 10% could be a more adequate predictive and prognostic marker than 1% in patients with non-metastatic breast cancer regardless of HER2 status. In 2020, the ASCO/CAP guideline was further updated, recommending reporting the samples with ER/PR expressions of 1%–9% as “low-positive” [9]. The 2023 St. Gallen International Consensus Conference report acknowledges the ongoing debate over the optimal ER threshold for hormone therapy in early breast cancer. Studies indicated a less favorable prognosis for patients with ER expression of 1 to 9% compared with those with ≥ 10% [10]. As a result, there are limited data on endocrine therapy benefits for breast cancers with 1%–10% of ER expression, keeping the optimal threshold in question.

As part of these efforts, it may be more desirable to use ER expression as a continuous variable rather than categorical by classifying ER status as positive (or negative). This enables assessing its influence on long-term clinical outcomes with more information retained. Modeling ER expression as a continuous variable with censored survival outcomes, however, requires a strong model assumption of ER with the hazard function. The hazard ratio should remain constant over time for any given unit increase in ER expression. Such an assumption is statistically necessary to analyze ER as a continuous variable for the risk of events such as disease recurrence or death in the Cox model. Unfortunately, the literature has noted that continuous ER expression data in HER2-negative patients do not satisfy the model assumption. Cutoff points of 1% or 10%, identified using tree-based methods based on the outcome of pCR, had to be used to categorize ER expression and evaluate its associations with long-term clinical outcomes [7].

We proposed a shape-restricted Cox regression model to overcome the limitation of statistical analysis in nonproportional hazards with a continuous variable and better assess the clinical impact of ER expression on survival outcomes. This flexible model does not require proportional hazards assumptions, and it can better capture dynamic clinical insights of a continuous variable, such as ER percentage, on survival outcomes [11].

In this study, we applied the newly proposed statistical method to overcome the limitation of the conventional proportional hazard model. This allows us to holistically estimate the effect of continuous ER expression on the long-term clinical endpoints, while also adjusting for other risk factors. This method provides a comprehensive view of the association between ER expression and long-term outcomes, helping to identify the proper threshold to define ER negativity. Thus, we can better identify triple-negative breast cancer among the HER2-negative subgroup. We also confirmed and compared the findings of the new method with those of conventional survival analysis methods.

## Patients and Methods

### Patient Selection

We retrospectively reviewed the Breast Medical Oncology Management System database at MD Anderson Cancer Center (MDA), and we identified patients with newly diagnosed stage II or III HER2-negative primary invasive breast cancer who received neoadjuvant chemotherapy followed by definitive surgery between June 1982 and June 2013 at MDA. Only patients with known ER and PR expression levels as continuous variables were included in this study. Patients who did not receive neoadjuvant chemotherapy had no curative intent surgery, or who received neoadjuvant endocrine therapy were excluded. This study was approved by the MD Anderson Institutional Review Board (protocol number PA14–0046).

### Statistical Methods

The clinical outcomes of interest included time to recurrence or death (TTR) and overall survival (OS). TTR was defined as the time from surgery to recurrence or death from breast cancer, whichever occurred first. OS was defined as the time from surgery to death from any cause, and patients who survived at the last follow-up were censored. The data were analyzed using the standard Cox regression model and the shape-restricted Cox regression model with a non-increasing constraint on ER [11]. Backward variable selection was used for multivariable Cox regression models with a p-value ≤ 0.05 as the inclusion limit. The proportional hazards (PH) assumption was tested for continuous ER expression using the standard Cox model. The Kaplan Meier curves for TTR and OS were plotted by the different levels of ER expression (0 ≤ ER < 1%, 1% ≤ ER < 10%, 10% ≤ ER < 20%, and ER ≥ 20%) for visualization, and the log-rank test was used for the comparisons of TTR or OS among the subgroups. The shape-restricted Cox regression model was used to estimate the effect of continuous ER expression on TTR and OS and to determine the potential thresholds for the clinical outcomes. Different from the standard Cox regression model, the shape-restricted Cox regression model does not restrict the linear effect of ER expression on survival outcomes, which overcomes the requirement of ER satisfying the proportional hazards assumption. All analyses were conducted using SAS 9.4 (SAS, Cary, NC, USA), R version 4.2.2 (R Foundation for Statistical Computing), and S-Plus 8.2 (TIBCO Software, Palo Alto, CA, USA) statistical software.

## Results

### Patient Demographic and Disease Characteristics

Of the 3853 female patients newly diagnosed with stage II or III HER2-negative primary invasive breast cancer for whom ER and PR continuous expression data were available, 3055 patients met the study criteria included in the analysis (**Supplementary Fig. S1**). To mitigate the issue of collinearity between ER and PR in the multivariable models, PR was not included in the multivariable models, since continuous ER and PR showed a strong correlation (Spearman correlation coefficient = 0.70; p < 0.0001). The median age was 49 years (range, 19–83 years), and 1579 patients (51.9%) were postmenopausal at the time of diagnosis, 62.7% were White, 15.2% were Black, 15.8% were Hispanic, 6.3% were Asian or other (Table 1). A total of 932 (30.5%) patients had tumors with ER < 1%, 171 (5.6%) had tumors with ER ≥ 1% but < 10% of ER expression, 67 (2.2%) had tumors with ER ≥ 10% but < 20% of ER expression, and 1885 (61.7%) had tumors with ≥ 20% ER expression. Of the 171 patients with 1% ≤ ER < 10% tumors, 43 (25.1%) received adjuvant endocrine therapy; of the 67 patients with 10% ≤ ER < 20% tumor, 54 (80.6%) received adjuvant endocrine therapy, and of the 1885 patients with ER ≥ 20% tumor, 1852 (98.2%) received adjuvant endocrine therapy. Most patients received radiation therapy (79.2%), and only 15.1% received adjuvant chemotherapy. The pCR rates for patients with different levels of ER expression were as follows: < 1% ER, 26.3%; 1% ≤ ER < 10%, 28.1%; 10% ≤ ER < 20%, 10.4%; ER ≥ 20%, 6.8%.

### Analysis Results of Standard and Shape-restricted Cox Regression Model

With a median follow-up of 3.9 years for OS (up to 14 years), 659 patients died, and 862 experienced recurrent disease. The standard Cox regression model was used to identify following factors significantly associated with poor TTR or OS: Black ethnicity, advanced nuclear grade, advanced tumor clinical stage, presence of lymphatic/vascular invasion, lack of radiation therapy, negative pCR status, and lower levels of continuous ER expression (Table 2). Continuous ER expression did not satisfy the proportional hazards assumption (p < 0.001), indicating a non-constant effect of ER expression on the risks of TTR and OS. The results nevertheless showed that lower ER expression was associated with an increased risk of death or recurrence. Unlike the standard Cox proportional hazard model, the shape-constrained Cox model allowed us to analyze the association of ER as a continuous variable with survival outcomes without assuming a specific model while considering the influence of other baseline risk factors. The estimated hazard function of death or recurrence-free death based on ER expression can also help identify the threshold value by assessing ER expression as a continuous variable, which is impossible with the standard Cox proportional hazards model.

After adjusting for other baseline covariates, the monotone shape-restricted function form on ER expression r(x) was modeled in the Cox regression framework to analyze the effect of ER expression as a continuous variable as follows:

$$\lambda (t|x,z)=\lambda (t)\exp\left(\beta 1\;\mathrm{age}\;+\;\beta 2\;\text{Asian}\;+\;\beta 3\;\text{Black}\;+\;\beta 4\;\text{Hispanic}+\beta 5\;\text{grade}\;+\;\;\beta 6\;\text{stage}\;\text{+}\;\beta 7\;\text{lymph}/\text{vascular}\;\text{invasion}\;+\;\beta 8\;\text{radiation}\;+\;\beta 9\;\text{pCR}\right)/r(x),$$

where x is the continuous ER expression, and z denotes the vector of other baseline covariates **(Supplementary Statistical Model).**

#### a) Time to Recurrence Outcome

**Figure 1:**
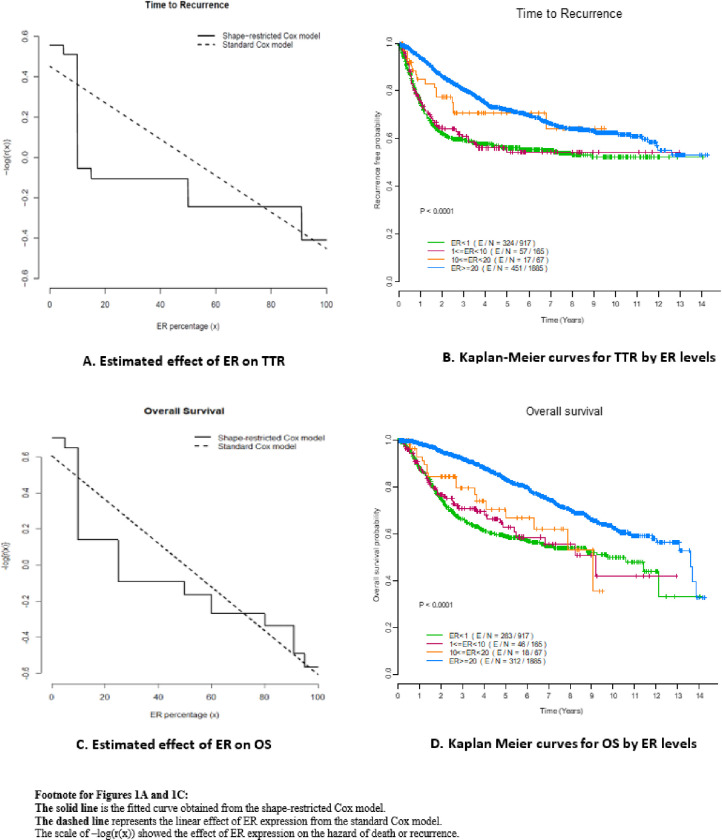
Estimated effects of ER and Kaplan-Meier curves by ER levels for TTR and OS

As shown in Table 2, grade III, stage III, positive lymphatic/vascular invasion, not receiving radiation therapy, or non-pCR were significantly associated with an increased risk of breast cancer recurrence or death in both standard and shape-restricted Cox models. The estimates of the HRs under the shape-restricted Cox regression model were close to those under the standard Cox regression model for these baseline covariates. Unlike in the standard Cox model, there is no constant HR (i.e., a linear effect of the continuous ER expression on the log of the cumulative hazard function) in the shape-restricted Cox model. This is because a nonlinear monotone function is provided to describe the association between ER expression and the hazard of recurrence, r(x). Figure 1A shows the estimated effect of ER expression on TTR using these two methods. The hazard curve, r(x), on the log transformation scale, modeled by ER expression using the shape-restricted model, showed a sharp decline on the risk of recurrence by an increase of ER expression from 0 to 10%. However, beyond 10% ER expression, the curve remained relatively flat, deviating considerably from the linear effect seen in the standard Cox model. While the convex shape of -log⁡(r(x)) confirmed the violation of the proportionality assumption of ER expression in TTR, the descriptive plot provided a holistic picture to help us better understand how continuous ER expression was associated with long-term survival outcomes. Such a descriptive plot provides a lead for a proper cutoff point when defining ER positivity. The shape-constrained nonparametric estimator implied that a cutoff of 10% for ER expression could be a proper threshold for identifying patients with good prognosis outcomes for HER-negative breast cancer patients.

The Kaplan Meier curve for TTR (Fig. 1B) confirmed the findings that a cutoff of 10% for ER expression in defining the positivity of ER based on TTR was appropriate. Patients with an ER expression above 10% had a significantly better TTR compared to those with an ER expression < 10% (log-rank p < 0.001). The TTR curves of ER < 1% and 1% ≤ ER < 10% were overlapping. After adjusting for age at diagnosis, race, nuclear grade, clinical stage, lymph vascular invasion, adjuvant radiation, and pCR, patients with ER expressions < 10% showed a significantly higher risk for breast cancer death or recurrence compared to those with ER expressions between 10% and 20% (HR = 2.181, 95% CI = 1.317–3.612, p = 0.002 for TTR). TTR was not significantly different between patients with 20% and above and patients with ER expressions between 10% and 20% (HR = 0.930, 95% CI = 0.561–1.542, p = 0.777 for TTR, Table 3).

#### b) Overall Survival Outcome

The effect of ER expression on the risk of death decreased sharply with increasing ER expression from 0–20% and then decreased much more slowly when ER expression increased from 20% (Fig. 1C). The shape-constrained Cox model could imply a cutoff of 20% for ER expression to be clinically relevant in predicting OS.

The Kaplan Meier curve (Fig. 1D) for OS among patients with different ER levels showed that patients with an ER expression above 20% had a significantly better OS compared to those with an ER expression < 20%, and there was no statistically significant difference between the other two ER categories (< 10% vs 10%–20%), as shown in the univariate analysis (Table 3). After adjusting for race, nuclear grade, clinical stage, lymph vascular invasion, adjuvant radiation, and pCR, patients with ER expressions above 20% showed a lower risk of death than patients with ER expressions 10%–20% (HR = 0.592, 95% CI = 0.360–0.974, p-value = 0.039 for OS; Table 3) and those with ER expressions < 10%. The multivariable model indicated that ER expression in OS exhibited two distinct levels, with an association that did not follow a proportional hazard function but showed a clear monotonic trend. As ER expression increases, the OS benefit also increases. However, the magnitude of the increase in survival benefit varies with different values of ER expression.

### TTR and OS Benefits from Adjuvant Endocrine Therapy

We performed subgroup survival analyses to investigate whether there was any survival benefit from the use of endocrine therapy among three patient subgroups defined by their ER expressions: 1% ≤ ER < 10%, 10% ≤ ER < 20%, and ER ≥ 20% (Table 4). There was no statistically significant benefit from receiving adjuvant endocrine therapy in patients with 1% ≤ ER < 10% for TTR and OS in the multivariable analysis. The results showed that among the 67 patients with 10% ≤ ER < 20% tumors, adjuvant endocrine therapy was not significantly associated with either TTR (HR = 0.645, 95% CI = 0.207–2.007, p = 0.449) or OS (HR = 0.429, 95% CI = 0.158–1.161, p = 0.096). As expected, among 1792 patients with ER ≥ 20% tumors, patients who received endocrine therapy had significantly improved TTR (HR = 0.113, 95% CI = 0.073–0.175, p < 0.001) or OS (HR = 0.199, 95% CI = 0.120–0.331, p < 0.001).

To further explore the survival benefit of adjuvant endocrine therapy among patients with different levels of ER expressions, we included interaction terms between adjuvant endocrine therapy and ER category (ER < 10% vs 10% ≤ ER < 20% vs ER ≥ 20%) in the multivariable Cox proportional hazards models for TTR and OS, respectively. The interactions between ER and adjuvant endocrine therapy were statistically significant in both the OS and TTR models when comparing ER > 20% with 10% ≤ ER < 20% (p = 0.002 for TTR; p = 0.034 for OS). This implies that the benefit of endocrine therapy on TTR and OS depends on the ER levels; adjuvant endocrine therapy had a significantly larger benefit on TTR and OS for patients with ER expressions above 20% compared with those with 10% ≤ ER < 20%.

## Discussion

By utilizing a flexible shape-restricted regression model, we can better present the holistic association between continuous ER expression and time to death or TTR after adjusting for other baseline covariates in patients with HER2-negative primary breast cancer who underwent neoadjuvant chemotherapy followed by curative surgery. For stage II/III HER2-negative patients, an ER threshold of 10% better predicts TTR and pCR status [7], whereas an ER threshold of > 20% seems to predict long-term OS better. Moreover, there was not statistically significant survival benefit to improve TTR from adjuvant hormonal therapy for patients with 1% ≤ ER < 10%.

Although it is widely agreed that ER expression monotonically decreases the risk of clinical prognosis for breast cancer patients, the ideal clinically meaningful cutoff value for ER expression for various clinical endpoints is still not well understood. The proposed monotone shape restriction inference is a natural method to handle such a challenge. In current clinical practice, 1% is used to determine the benefit for endocrine therapy in both adjuvant and metastatic settings. We previously showed that a 10% ER cutoff may be more effective in predicting the pCR status after neoadjuvant chemotherapy and the benefits of adjuvant endocrine therapy [7]. However, there is no consensus supported by validated studies. One of the challenges in identifying the desirable cutoff is the non-feasibility of designing prospective clinical trials targeting such a small patient population, which requires a long follow-up time. Therefore, reanalyzing existing data using a better statistical method is the key to solving this problem.

In this study, we used the newly proposed statistical method, a flexible monotone-shaped Cox regression model, to estimate possibly non-constant effect of continuous ER expression on long-term clinical endpoints with adjustment for other risk factors. The new model can enhance the interpretation of the ER effect and provide a global look at how continuous ER expression is associated with long-term outcomes without assuming a linear effect on hazards. The estimated monotone function of ER expression against the hazard/risk of prognosis could indicate reliable cutoff points for different clinical outcomes and provide more meaningful clinical insights to assess its effect on long-term overall survival.

By using this method, our results reported that 10% is the desirable cutoff to predict TTR, which is consistent with our previous publication [7] and our current data will be additional evidence to support the idea that 10% rather than 1% is a better cut-off in neoadjuvant and adjuvant setting. To predict OS, ER of 20% and higher was found to be a strong indicator for assessing treatment effects of macrometastatic diseases. Although we did not observe the benefits of endocrine therapy in patients with an ER 10%–20% on TTR in the subgroup analysis, this could be due to the small sample size.

There are several limitations in the current study. The study period was from 1982 to 2013, and today, there are more standard-of-care therapies for hormone receptor-positive HER2-negative breast cancer in adjuvant and metastatic settings, such as adjuvant abemacilib [12] and alpelisib [13]. This may have affected our results. Of note, 54 of the 67 patients with 10% ≤ ER < 20% tumors (80.6%) and 1852 of the 1885 patients with ER ≥ 20% tumors (98.2%) received adjuvant endocrine therapy. During the study, adjuvant endocrine therapy was the standard of care for patients with an ER of > 10%. Although the number is small, those who did not receive adjuvant endocrine therapy must have had reasons (e.g., severe osteoporosis, thrombosis, uterine cancer, etc.). Additionally, we were not able to assess the HER-2 low group separately. The results of the DESTINY-Breast04 trial showed significant efficacy of HER2 target therapy with trastuzumab deruxtecan in patients with HER2-low expressing breast cancer, providing another insight into the interpretation of the current study [14]. This could be due to bystander effect to the tumors surrounding those with at least some HER2 expression. Also, there is a possibility that HER2-low disease has a completely distinct biology or a unique tumor microenvironment.

Our findings confirmed that 10% expression of ER is an ideal cut-off to predict TTR and that patients with ER < 10% do not have a long-term prognosis benefit of adjuvant endocrine therapy. This offers practical insights to guide physicians in balancing the benefits and risks of adjuvant endocrine therapy for ER-positive patients with an ER 1–10% disease. This would be particularly useful in several challenging clinical situations, such as whether endocrine therapy discontinuation is appropriate when patients struggle to take their medication due to toxicities or whether patients need to continue endocrine therapy for over five years. For example, physicians may feel more comfortable discontinuing adjuvant endocrine therapy in patients with an ER 1–10% when patients suffer from treatment-related side effects such as severe arthralgia and menopause symptoms. The study findings underscore the potential of tailoring treatment decisions based on ER expression levels to enable more informed choices, particularly in cases involving severe adverse events due to endocrine therapy.

## Conclusions

The shape-restricted Cox regression model holistically demonstrates the dynamic association between continuous ER expression and long-term clinical outcomes in breast cancer and assists in selecting the desirable cutoff value for ER expression to optimize endocrine therapy use. Based on the adjusted shape-restricted Cox regression model, the results suggest an ER expression threshold of 10% for predicting TTR. The findings could assist physicians in effectively weighing the benefits and risks of adjuvant endocrine therapy for patients with 1% < ER < 10% disease, particularly in cases involving severe adverse events in metastatic settings. Further validation through retrospective or prospective studies is necessary to confirm these findings, thereby contributing to enhanced patient care and treatment strategies.

## Figures and Tables

**Table 1.** Patient characteristics and treatment by ER

Variable	levels	Total	ER < 1%	1% ≤ ER < 10%	10% ≤ ER < 20%	ER ≥ 20%	p
		n = 3055 (100%)	n = 932 (30.5%)	n = 171 (5.6%)	n = 67 (2.2%)	n = 1885 (61.7%)	
Age, median (range), years		49 (19–83)	49 (22–83)	49 (19–77)	49 (30–74)	50 (22–83)	0.430
BMI, median (range), kg/m2		27.7 (14.5–65.9)	29.0 (14.5–56.4)	30 (17.7–61.3)	29.6 (16.2–48.7)	28.9 (15.5–65.9)	0.043
Race/ethnicity							
	White	1915 (62.7%)	565 (60.6%)	97 (56.7%)	33 (49.3%)	1220 (64.7%)	<0.001
	Black	463 (15.2%)	197 (21.1%)	35 (20.5%)	15 (22.4%)	216 (11.5%)	
	Hispanic	483 (15.8%)	128 (13.7%)	25 (14.6%)	15 (22.4%)	315 (16.7%)	
	Asian/others	194 (6.3%)	42 (4.5%)	14 (8.2%)	4 (6%)	134 (7.1%)	
Menopausal status							
	Premenopausal	1461 (48.1%)	438 (47.2%)	84 (49.1%)	38 (56.7%)	901 (48.1 %)	0.506
	Postmenopausal	1579 (51.9%)	489 (52.8%)	87 (50.9%)	29 (43.3%)	974 (51.9%)	
	Unknown	15	5			10	
Histology							
	Ductal	2577 (85.2%)	851 (93.9%)	156 (94.5%)	60 (89.6%)	1510 (80.1 %)	<0.001
	Lobular	231 (7.6%)	15 (1.7%)	2 (1.2%)	6 (9%)	208 (11 %)	
	Others	215 (7.1%)	40 (4.4%)	7 (4.2%)	1 (1.5%)	167 (8.9%)	
	Unknown	32	26	6			
Clinical stage							
	II	1726 (56.5%)	511 (54.8%)	99 (57.9%)	36 (53.7%)	1080 (57.3%)	0.596
	III	1329 (43.5%)	421 (45.2%)	72 (42.1%)	31 (46.3%)	805 (42.7%)	
Nuclear grade							
	I/II	1163 (39.2%)	91 (10.1%)	19 (11.4%)	15 (23.4%)	1038 (56.7%)	<0.001
	III	1803 (60.8%)	813 (89.9%)	148 (88.6%)	49 (76.6%)	793 (43.3%)	
	Unknown	89	28	4	3	54	
ER, continuous, mean+/−SD		51.9 +/− 43.2	0 +/− 0.2	3.7 +/− 2.3	11.5 +/− 2.4	83.3 +/− 20.8	
PR, continuous, mean+/−SD		31.6 +/− 38.2	2.4 +/− 11.3	4.6 +/− 12.5	10.8 +/− 22.8	+/− 38.2	<0.001
Lymph vascular invasion							
	Negative	2030 (68.9%)	631 (71.3%)	124 (77.5%)	45 (69.2%)	1230 (67%)	0.012
	Positive	915 (31.1%)	254 (28.7%)	36 (22.5%)	20 (30.8%)	605 (33%)	
	Unknown	110	47	11	2	50	
Skin involvement							
	Negative	2762 (97.1%)	848 (96.6%)	149 (98%)	61 (100%)	1704 (97.1 %)	0.481
	Positive	83 (2.9%)	30 (3.4%)	3 (2%)	0 (0%)	50 (2.9%)	
	Unknown	210	54	19	6	131	
Adjuvant chemotherapy							
	No	2594 (84.9%)	770 (82.6%)	155 (90.6%)	58 (86.6%)	1611 (85.5%)	0.032
	Yes	461 (15.1%)	162 (17.4%)	16 (9.4%)	9 (13.4%)	274 (14.5%)	
Adjuvant hormonal therapy							
	No	1020 (33.4%)	846 (90.8%)	128 (74.9%)	13 (19.4%)	33 (1.8%)	<0.001
	Yes	2035 (66.6%)	86 (9.2%)	43 (25.1%)	54 (80.6%)	1852 (98.2%)	
Adjuvant radiation							
	No	636 (20.8%)	237 (25.4%)	50 (29.2%)	14 (20.9%)	335 (17.8%)	<0.001
	Yes	2419 (79.2%)	695 (74.6%)	121 (70.8%)	53 (79.1%)	1550 (82.2%)	
pCR							
	No	2626 (86.0%)	687 (73.7%)	123 (71.9%)	60 (89.6%)	1756 (93.2%)	<0.001
	Yes	429 (14.0%)	245 (26.3%)	48 (28.1%)	7(10.4%)	129 (6.8%)	

**Table 2:** Estimated regression coefficients with standard errors (SEs) and hazard ratios (HRs) from the standard Cox model and shape-restricted (SR) Cox model for TTR and OS

	**Standard Cox Model for TTR**				**Shape-restricted Cox model for TTR**			
	**Coeff.**	**SE**	**HR (95% CI)**	**p (Cox)**	**Coeff.**	**SE** *	**HR (95% CI)** *	P* **(SR Cox)**
**Age at diagnosis** (every 1-year increase)	−0.018	0.003	0.982 (0.975–0.988)	<0.001	−0.019	0.004	0.981 (0.975–0.988)	<0.001
**Race** (Asian vs White)	−0.290	0.169	0.748 (0.537–1.042)	0.086	−0.312	0.184	0.732 (0.510–1.051)	0.091
(Black vs White)	0.030	0.100	1.031 (0.848–1.253)	0.762	0.027	0.102	1.028 (0.842–1.254)	0.786
(Hispanic vs White)	−0.276	0.110	0.759 (0.612–0.941)	0.012	−0.273	0.116	0.761 (0.606–0.955)	0.019
**Nuclear grade** (I/II vs III)	−0.361	0.085	0.697 (0.589–0.823)	<0.001	−0.342	0.084	0.711 (0.602–0.838)	<0.001
**Clinical stage** (II vs III)	−0.829	0.078	0.436 (0.374–0.509)	<0.001	−0.831	0.083	0.436 (0.370–0.512)	<0.001
**Lymph vascular invasion** (Pos vs Neg)	0.715	0.075	2.045 (1.766–2.368)	<0.001	0.718	0.078	2.051 (1.761–2.389)	<0.001
**Adjuvant radiation** (Yes vs No)	−0.570	0.093	0.565(0.472–0.678)	<0.001	−0.578	0.103	0.561 (0.459–0.686)	<0.001
**pCR** (Yes vs No)	−1.463	0.177	0.231 (0.164–0.327)	<0.001	−1.511	0.188	0.221 (0.153–0.319)	<0.001
**Estrogen receptor** ^#^, continuous	−0.009	0.001	0.991 (0.989–0.993)	<0.001				
	**Standard Cox Model for OS**				**Shape-restricted Cox model for OS**			
	**Coeff.**	**SE**	**HR (95% CI)**	**P (Cox)**	**Coeff.**	**SE** *	**HR (95% CI)** *	P* **(SR Cox)**
**Race** (Asian vs White)	−0.602	0.248	0.548 (0.337–0.891)	0.015	−0.620	0.278	0.538 (0.312–0.927)	0.026
(Black vs White)	0.281	0.107	1.326 (1.075–1.635)	0.008	0.277	0.134	1.319 (1.015–1.715)	0.039
(Hispanic vs White)	−0.081	0.121	0.921 (0.727–1.167)	0.497	−0.079	0.125	0.924 (0.723–1.180)	0.526
**Nuclear grade** (I/II vs III)	−0.351	0.097	0.704 (0.581–0.852)	<0.001	−0.333	0.098	0.717 (0.592–0.868)	0.001
**Clinical stage** (II vs III)	−0.917	0.09	0.400 (0.335–0.476)	<0.001	−0.919	0.099	0.399 (0.329–0.485)	<0.001
**Lymph vascular invasion** (Pos vs Neg)	0.615	0.085	1.850 (1.566–2.183)	<0.001	0.624	0.088	1.866 (1.570–2.218)	<0.001
**Adjuvant radiation** (Yes vs No)	−0.582	0.102	0.559 (0.457–0.682)	<0.001	−0.581	0.114	0.559 (0.447–0.700)	<0.001
**pCR** (Yes vs No)	−1.655	0.216	0.191 (0.125–0.292)	<0.001	−1.695	0.223	0.184 (0.118–0.285)	<0.001
**Estrogen receptor** ^#^, continuous	−0.012	0.001	0.988 (0.986–0.990)	<0.001				

# The continuous estrogen receptor (ER) expression did not satisfy the proportional hazards assumption (p< 0.001).

* The SEs were obtained using the bootstrap method with 200 replicates. The 95% CIs and p-values were calculated based on the bootstrap SEs.

**Table 3:** ER effect in univariate and multivariable Cox proportional hazards model for TTR and OS

ER Level	ER effect on TTR		ER effect on OS	
	HR (95% CI)	P	HR (95% CI)	P
**Univariate**				
**0% ≤ ER < 10%**	1.653 (1.017–2.687)	0.043	1.321 (0.822–2.123)	0.250
**% ≤ ER < 20%**	Reference		Reference	
**ER ≥ 20%**	0.803 (0.495–1.304)	0.375	0.496 (0.308–0.798)	0.004
**Multivariable** *				
**0% ≤ ER < 10%**	2.181 (1.317–3.612)	0.002	1.741 (1.063–2.852)	0.028
**10% ≤ ER < 20%**	Reference		Reference	
**ER ≥ 20%**	0.930 (0.561–1.542)	0.777	0.592 (0.360–0.974)	0.039

* With the adjustment for age at diagnosis (for TTR only), race, nuclear grade, clinical stage, lymph vascular invasion, adjuvant radiation, and pCR.

**Table 4:** Subgroup analysis to evaluate adjuvant hormonal therapy effect in multivariable Cox proportional hazards models for TTR and OS

		Hormonal Therapy (Yes vs No)		Other covariates included *
Event	Subgroup	HR (95% CI)	P	
**TTR**				
	**1% ≤ ER < 10%**	0.877 (0.481–1.600)	0.668	Clinical stage, lymph vascular invasion, and pCR
	**(n = 157)**			
	**10% ≤ ER < 20%**	0.645 (0.207–2.007)	0.449	None^#^
	**(n = 67)**			
	**ER ≥ 20%**	0.113 (0.073–0.175	<.001	Age at diagnosis, race, nuclear grade, clinical stage, lymph vascular invasion, adjuvant radiation, and pCR
	**(n = 1792)**			
**OS**				
	**1% ≤ ER < 10%**	0.698 (0.3371.446)	0.333	Clinical stage, lymph vascular invasion, adjuvant radiation, and pCR
	**(n = 157)**			
	**10% ≤ ER < 20%**	0.429 (0.158–1.161)	0.096	None^#^
	**(n = 67)**			
	**ER ≥ 20%**	0.199 (0.120–0.331)	<.001	Race, nuclear grade, clinical stage, lymph vascular invasion, adjuvant radiation, and pCR
	**(n = 1792)**			

* Only covariates with significant association with OS or TTR (p value 0.05 used as the cutoff) were included in the multivariable Cox proportional hazards models.

# None of the covariates were statistically significantly associated with OS or TTR after adjusting for hormonal therapy.

## Data Availability

The data underlying this article cannot be directly shared due to the privacy of individuals that participated in the study. Access to these data can be provided to researchers under certain circumstances, pending approval by the Institutional Review Board of The University of Texas MD Anderson Cancer Center.
